# Spray-Congealing and Wet-Sieving as Alternative Processes for Engineering of Inhalation Carrier Particles: Comparison of Surface Properties, Blending and ***In Vitro*** Performance

**DOI:** 10.1007/s11095-021-03061-5

**Published:** 2021-06-10

**Authors:** Joana T. Pinto, Sarah Zellnitz, Tomaso Guidi, Francesca Schiaretti, Hartmuth Schroettner, Amrit Paudel

**Affiliations:** 1 Research Center Pharmaceutical Engineering GmbH, Inffeldgasse 13, 8010 Graz, Austria; 2 Chiesi Farmaceutici S.p.A., R&D Department, Largo F. Belloli 11/A, 43122 Parma, Italy; 3 Austrian Centre for Electron Microscopy and Nanoanalysis, TU Graz, Steyrergasse 17/III, 8010 Graz, Austria; 4 Institute of Process and Particle Engineering, Graz University of Technology, Inffeldgasse 13, 8010 Graz, Austria

**Keywords:** D-mannitol, dry powder inhaler, particle engineering, spray-congealing, wet-sieving

## Abstract

**Purpose:**

Traditionally, α-lactose monohydrate is the carrier of choice in dry powder inhaler (DPI) formulations. Nonetheless, other sugars, such as D-mannitol, have emerged as potential alternatives. Herein, we explored different particle engineering processes to produce D-mannitol carriers for inhaled delivery.

**Methods:**

Wet-sieving and spray-congealing were employed as innovative techniques to evaluate the impact of engineering on the particle properties of D-mannitol. To that end, the resulting powders were characterized concerning their solid-state, micromeritics and flowability. Afterwards, the engineered carrier particles were blended with inhalable size beclomethasone dipropionate to form low dose (1 wt%) DPI formulations. The *in vitro* aerosolization performance was evaluated using the NEXThaler®, a reservoir multi-dose device.

**Results:**

Wet-sieving generated D-mannitol particles with a narrow particle size distribution and spray-congealing free-flowing spherical particles. The more uniform pumice particles with deep voids and clefts of wet-sieved D-mannitol (Pearl300_WS) were beneficial to drug aerosolization, only when used in combination with a ternary agent (10 wt% of ‘Preblend’). When compared to the starting material, the spray-congealed D-mannitol has shown to be promising in terms of the relative increase of the fine particle fraction of the drug (around 100%), when used without the addition of ternary agents.

**Conclusions:**

The wet-sieving process and the related aerosolization performance are strongly dependent on the topography and structure of the starting material. Spray-congealing, has shown to be a potential process for generating smooth spherical particles of D-mannitol that enhance the *in vitro* aerosolization performance in binary blends of the carrier with a low drug dose.

**Supplementary Information:**

The online version contains supplementary material available at 10.1007/s11095-021-03061-5.

## Introduction

In dry powder inhalation (DPI), drugs are delivered as solid particles to the lung. In order to reach the lower respiratory airways, the drug particles have to be in the size range of 0.5/1.0 to 5.0 μm (1). Due to their very large surface to volume ratio, the particles in the aforementioned size range, are cohesive and possess poor powder flow. The flowability and dispersion of the inhaled active pharmaceutical ingredient (API) particles can be improved by specifically tailoring their properties (e.g., to free-flowing large porous particles) or blending with excipients. Traditionally, the drug particles are blended with coarse excipient ones (Dv_0.5_ ≥ 20 μm) in a practice known as adhesive mixing. Adhesive mixing aims to attach the smaller API particles onto the surface of larger excipient ones. Likewise, the excipient functions as a ‘carrier’ and such formulations are called carrier-based DPI formulations.

α-lactose monohydrate (α-LH) particles are typical DPI carriers presenting a tomahawk shape and a mean particle size (Dv_0.5_) between 50 μm and 250 μm, depending on the device used. α-LH can be blended solely with the API, forming binary blends or can be mixed with the drug and other excipient particles that are added in order to improve the aerosolization. These excipient particles, presenting a size smaller than the coarse carrier, are usually known as fines. Excipient fines are usually magnesium stearate or lactose particles alone or in a mixture. Fine particles are known to aid the performance of DPIs by a variety of mechanisms (2–4). Although α-LH has been typically used as a carrier, its complex solid state, encompassing 2 anomeric forms, 2 anhydrous polymorphs and 1 hydrate of its α-anomer as well as an amorphous phase makes engineering of lactose particles quite challenging. Moreover, high temperature engineering processes cannot be applied to lactose due to its degradation upon melting. Thus, D-mannitol, a sugar approved for inhalation (5), is regarded as a good alternative to lactose. D-mannitol crystals exist as two polymorphs (the α- and β-forms) with very similar thermodynamic behavior that are monotropically related, and a metastable form enantiotropically (δ-form) related to the β-polymorph. Although, an amorphous phase and a hemi-hydrate of the β-polymorph are known, these forms cannot be easily obtained under ambient conditions. Moreover, D-mannitol does not degrade upon melting offering the possibility to process it at high temperatures. Thus, D-mannitol has been extensively studied as a potential carrier for DPIs in recent years (6–9). In 2017, Merck has started commercializing a grade of D-mannitol intended to be used as a carrier in DPI formulations (10).

During inhalation, carrier particles will impact the upper respiratory tract and get swallowed or expectorated. Thus, although drug particles need to attach with sufficient force to the carrier surface to be properly handled during manufacturing, and in particular to facilitate dose metering, the attachment also has to be loose enough to allow drug detachment during inhalation. DPI formulations are commonly delivered using inhalation devices that rely on the inspiratory forces of patients to fluidize the powder and promote detachment. Hence, when designing excipient particles for inhalation a combination of factors related to the carrier and API combination as well as type of inhalation device and its dependency on the patient inspiratory force, should be considered (1). Often, excipient particles lack the most adequate characteristics when obtained from primary manufacturing and have to be further engineered. Therefore, in the present work, we aim to explore innovative engineering strategies that allow the production of D-mannitol particles, which have adequate characteristics, to be successfully applied as carriers for DPI.

Wet-sieving, commonly used in soil analysis (11), and previously used by Adi *et al*. to fractionate lactose particles intended for inhalation (12), was selected to engineer D-mannitol. During wet-sieving, powder particles are washed with an anti-solvent and fractionated. Thus, wet-sieving was used for removing the variable amount of fine particles present in the raw excipient powder. Spray-congealing (also known as spray-chilling or spray-cooling), which is a melt-based technique able to produce well-defined spherical particles (13), was also applied to engineer D-mannitol. In this process, molten D-mannitol was atomized into droplets through a bi-fluid nozzle and sprayed into a cooling chamber where the melt quickly solidified forming solid microparticles. The technique was selected due to its known ability to produce free-flowing particles with a diameter between 50 and 500 μm.

To evaluate the impact of engineering on particle properties, D-mannitol powders were characterized concerning their solid-state, micromeritics and flowability. Afterwards, D-mannitol was blended (in binary and ternary mixtures) in low doses (1 wt%) with an inhaled corticosteroid (i.e., beclomethasone dipropionate). The obtained blends were tested in combination with a reservoir inhaler (NEXThaler®) and the aerosolization performance of the distinct particles of D-mannitol was evaluated, accordingly. Whenever relevant, the performance of the D-mannitol particles was compared to that of α-LH engineered by the same techniques. Eventually, it was possible to understand the applicability of wet-sieving and spray-congealing in producing novel D-mannitol carriers for DPI delivery.

## Materials and Methods

### Materials

D-mannitol (Pearlitol® 300 DC) was purchased from Roquette (France) and α-LH (CapsuLac® 60 and InhaLac® 120) was obtained from Meggle (Germany). The model API, beclomethasone dipropionate (BDP) with a mean particle size of 1.05 μm was provided by Chiesi Pharmaceutici S.p.A, Italy. Magnesium Stearate (MgSt) as well as acetone were purchased from Merck KGaA, Germany.

### Particle Engineering

#### Surface Processing Via Wet-Sieving

**Fig. 1:**
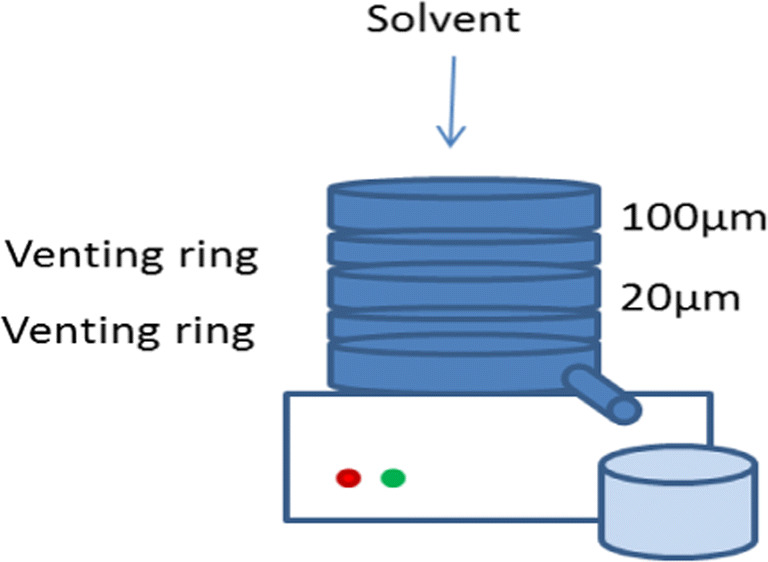
Experimental set-up used for wet sieving.

The experimental set-up used for wet-sieving in the present study was based on the work by Robertson *et al*. (11). A standard vibratory sieve shaker (AS200, Retsch, Germany), as schematically shown in Fig. 1 was used to wet-sieve the particles of D-mannitol.

Before wet-sieving, D-mannitol and acetone (anti-solvent) were mixed in a 1:2 wt% ratio and placed in an ultrasonic bath for 5 min. The resulting mixture was transferred to a 100 μm sieve (top of the tower) and the vibration intensity was set to 0.2. The wet-sieving of the powder was then carried out by consecutively rinsing the sample with acetone (400 ml for 20 g of powder) as the tower was vibrating. When the process was finished, the washed material was transferred to a filter paper and placed under a fume hood for 24 h in order to let the material dry. α-LH (CapsuLac®60) was also engineered using the same procedure as a control/reference for comparison.

#### Spray-Congealing

**Fig. 2:**
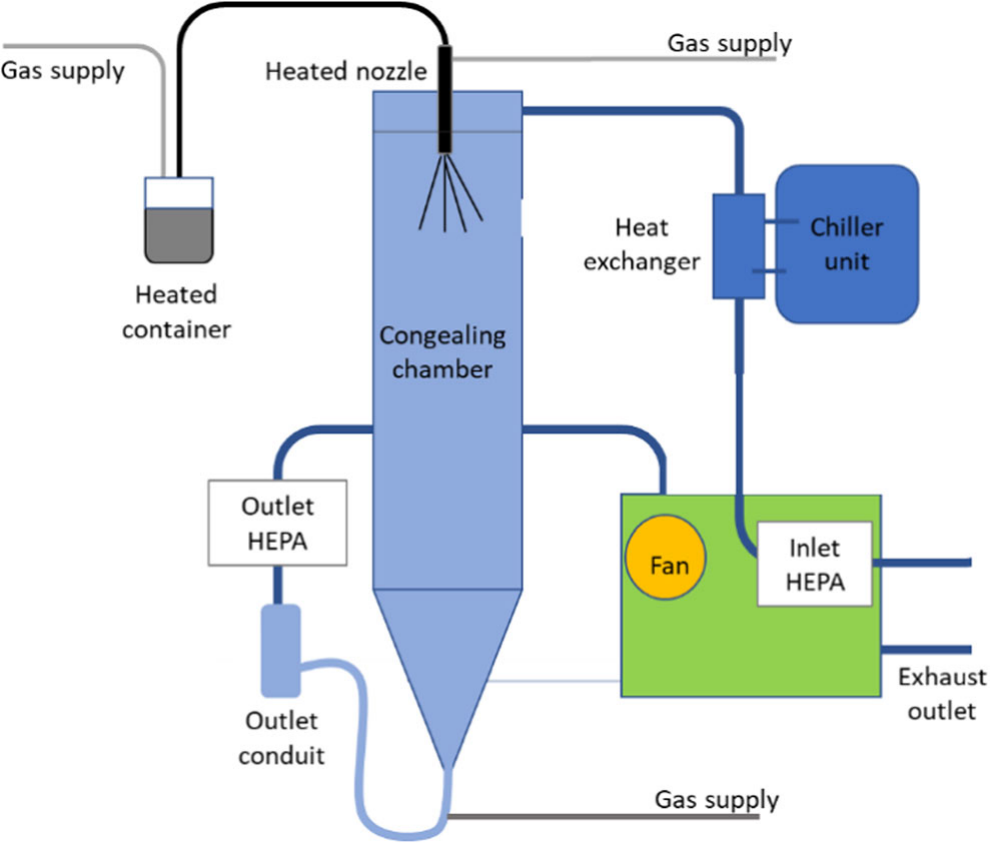
Schematic of spray-congealing set-up.

The particles of D-mannitol were also engineered via spray-congealing (4 M8-TriX spray-congealer, ProCepT, Belgium). In this process, D-mannitol was first placed in an oven at 190°C and once a melt was obtained this was transferred to a heated vessel kept at 200°C. The molten fluid was pumped at 20.0 ± 1.4 g/min from the vessel to a heated bi-fluid nozzle (diameter 1.2 mm) and atomized (0.44 bar) into a glass tower chilled at −10°C, using a nitrogen flow of 0.6 m^3^/min. The solidified particles were separated from the air stream using a cyclone set at a pressure drop of 13 mbar and collected in a glass vessel at 13°C (Fig. 2).

### Characterization of the Engineered Powders

#### Scanning Electron Microscopy (SEM)

The powder samples were observed using scanning electron microscopy (SEM). For this, the particles were sputtered with gold-palladium and then examined using a Zeiss Ultra 55 scanning microscope (Zeiss, Germany), operating at 5 kV.

#### Particle Size Distribution

The particle size distribution (PSD) was determined using laser light diffraction (HELOS/KR, Sympatec GmbH, Germany). A dry dispersing system (Rodos, Sympatec) and a vibrating chute (Vibri, Sympatec) were used for powder dispersion. The powders were analyzed using a primary dispersion pressure of 1.0 bar and using the R2 (0.45–87.50 μm) and R5 (4.5–875.0 μm) optical modes. The measurements were triggered once an optical concentration of 0.5% was achieved. The resulting volumetric particle size distributions were calculated and analyzed using Windox 5 software (Sympatec GmbH, Germany).

#### Particle Solid-State

The solid-state of the particles was characterized by differential scanning calorimetry (DSC 204F1 Phoenix®, Netzsch GmbH, Germany) and wide-angle X-ray scattering (WAXS, S3-MICRO camera, Bruker AXS GmbH, Germany).

For the DSC analysis, 10–12 mg sample were weighed into an aluminum pan and crimped with a pierced lid. The samples were heated from 25 to 200°C at a rate of 10°C/min using pure nitrogen as purging gas at a flow rate of 20 mL/min. The DSC data analysis was conducted with Proteus Thermal Analysis software (Netzsch GmbH, Germany).

For the WAXS analysis, the powder samples were filled into 2 mm glass capillaries and analyzed under constant rotation (9 rpm) between the angular range of 17 and 27° 2θ during 600 s at 30 counts/s (22 ± 2°C).

#### Determination of the Specific Surface Area and Bulk Roughness

Nitrogen gas adsorption (TriStar II 3020, Micromeretics, USA) was used to determine the specific surface area of the powders. Before analysis, the samples were treated under vacuum (VacPrep 061, Micromeritics, USA) overnight at room temperature (22 ± 2°C). A 5-point analysis was performed using a nitrogen relative pressure (p/p_0_) between 0.06–0.20 and the Brunauer – Emmett – Teller (BET) adsorption theory was used to calculate the specific surface area (SSA_BET_).

The bulk roughness (BR) of the different D-mannitol particles was calculated as the ratio between the SSA_BET_ and the theoretical specific surface area (SSA_sv_) calculated based on the particle size volume-distribution assuming spherical particles (Eq.1) (14, 15).

1 $$ {\mathrm{SSA}}_{\mathrm{SV}}=\frac{6\ }{{\mathrm{D}}_{\mathrm{sv}}\times \uprho} $$

The D_sv_ is the Sauter mean diameter determined by laser diffraction as described above in the relevant section and ρ is the true density of the particles. The true density was calculated as the ratio between the mass of particles and their respective volume. The particle volume was determined by helium pycnometry (AccuPyc II 1340, Micromeritics, USA), using 20 purges at 19.5 psi with an equilibration rate of 0.0050 psi/min.

#### Powder Flowability

The flowability of the powders was measured using a FT4 Powder Rheometer (Freeman Technology, UK). An appropriate amount of sample was placed in a 1 ml cell in order to fill it. A standard conditioning cycle was applied using a 23.5 mm blade moving down a helical path in order to generate a uniform low packing powder bed. Afterwards, the surplus of powder was removed by splitting the cell and the blade was replaced by a 24 mm shear cell. The powder was pre-consolidated at 9 kPa and then sheared at 7, 6, 5, 4, and 3 kPa. The flowability parameters of the powders were obtained from the Mohr’s stress circles. The major principal (δ_1_) and unconfined yield (δ_c_) stresses are the highest values at which the larger and smaller Mohr’s stress intercept the x axis. The cohesion (Coh) is the point of intersection of the yield locus (normal stress = 0). The flow function coefficient (ffc) is the ratio between the major principal stress and the unconfined yield stress. The angle of internal friction (AIF_eff_) was determined from the effective yield locus.

### Evaluation of Powder Performance for Dry Powder Inhalation

To evaluate the applicability of the D-mannitol powders as carriers for inhalation, the particles were blended with BDP (Dv_0.5_ = 1.05 μm), yielding low load (1 wt%) adhesive blends that were tested concerning their aerosolization performance. For comparison, α-LH particles (CapsuLac®60, Meggle, Germany) were blended and tested under similar conditions.

#### Preparation of the Adhesive Mixtures

To obtain 20 g binary mixture of API and carrier, 200 mg of BDP were weighted and placed in a 50 ml glass bottle between two even layers of the excipient (19.8 g). The glass bottle was place in a Turbula blender TC2 (Willy A. Bachofen Maschinenfabrik, Switzerland) and mixed for 2 h at 32 rpm. The obtained blend was sieved through a 400 μm mesh and mixed again for 30 min at 32 rpm. To yield 20 g of ternary mixture of the carrier, API and 0.2 wt% magnesium stearate (MgSt) or 10 wt% ‘Preblend’ (composed of InhaLac® 120 and 0.2% MgSt), 40 mg of MgSt or 2 g of ‘Preblend’ were weighted and placed in 50 ml glass bottles between two even layers of D-mannitol (19.96 and 18.00 g of carrier, respectively). The glass bottles were placed in a Turbula blender TC2 (Willy A. Bachofen Maschinenfabrik, Switzerland) and mixed for 4 h at 32 rpm. Once a mixture of carrier and MgSt or ‘Preblend’ was obtained, the powders were blended with 1 wt% BDP following the same procedure as for the binary blends.

#### Mixing Homogeneity

The mixing homogeneity was determined by taking 10 samples of about 20 mg from distinct zones of the bulk powder blends. The samples were dissolved in a small amount of methanol and after that 100 ml of a mixture of methanol and water (70:30 vol%) were added and the samples were placed in an ultrasonic bath for 5 to 10 min in order to completely dissolve the API. The API content in each sample was analyzed via high performance liquid chromatography (described in the relevant section below) and the mixing homogeneity expressed as the relative standard deviation (RSD) from the mean drug content.

#### Shot Weight Consistency

Samples of binary and ternary blends (1.5 g) were manually filled into NEXThaler® devices (Chiesi, Italy) and the filled inhalers conditioned overnight in desiccators at 60% RH. The shot weight consistency was analyzed by firing the inhalers 10 times into a DUSA (Dosage Unit Sampling Apparatus) and determining the mass delivered via each actuation using the weight difference, before and after emission. Between each emission the inhalers were discharge using antistatic bars. Likewise, the mean shot weight and respective standard deviation were calculated based on the weight of 10 shots and the delivered mass consistency evaluated, accordingly.

#### Evaluation of the Aerodynamic Performance

The aerodynamic performance of binary and ternary DPI blends was assessed via next generation impactor (NGI, Copley Scientific, UK) experiments (16). To avoid particle bouncing, the stages of the impactor were coated with 2 vol% Tween 20 in ethanol prior to analysis. For each NGI experiment, one shot of the conditioned (at 60% RH) NEXThaler® was discharged. The flow rate was set to 60 L/min in order to reach a pressure drop of 4 kPa over the inhaler and the flow was applied for 4 s in order to ensure that 4 L of air were drawn via the mouthpiece over the inhaler. The drug content in each part of the impactor was quantified using a validated high performance liquid chromatography method (following section) and for each inhaler three NGI experiments were performed. The BDP *in vitro* deposition profile, fine particle fraction (FPF), fine particle dose (FPD), and the emitted dose (ED) were calculated and compared.

#### High Performance Liquid Chromatography (HPLC)

BDP was quantified via HPLC using a Waters Alliance 2695 HPLC system equipped with a 2996 photodiode array detector. A Waters Atlantis dC18 column (150 × 3.9 mm; 3 μm) was used as stationary phase and mobile phase was composed of acetonitrile/0.02 M sodium di-hydrogen phosphate monohydrate set to pH 3.0 (70:30 vol%). The system was operated in isocratic mode at a flow rate of 1 mL/min and a column temperature of 40°C. The injection volume was 50 μL and run time was 6.0 min, whereas the retention time of BDP was approximately 4.2 min. The UV-detection was conducted at a wavelength of 238 nm.

## Results and Discussion

### Particle Engineering by Wet-Sieving

**Fig. 3:**
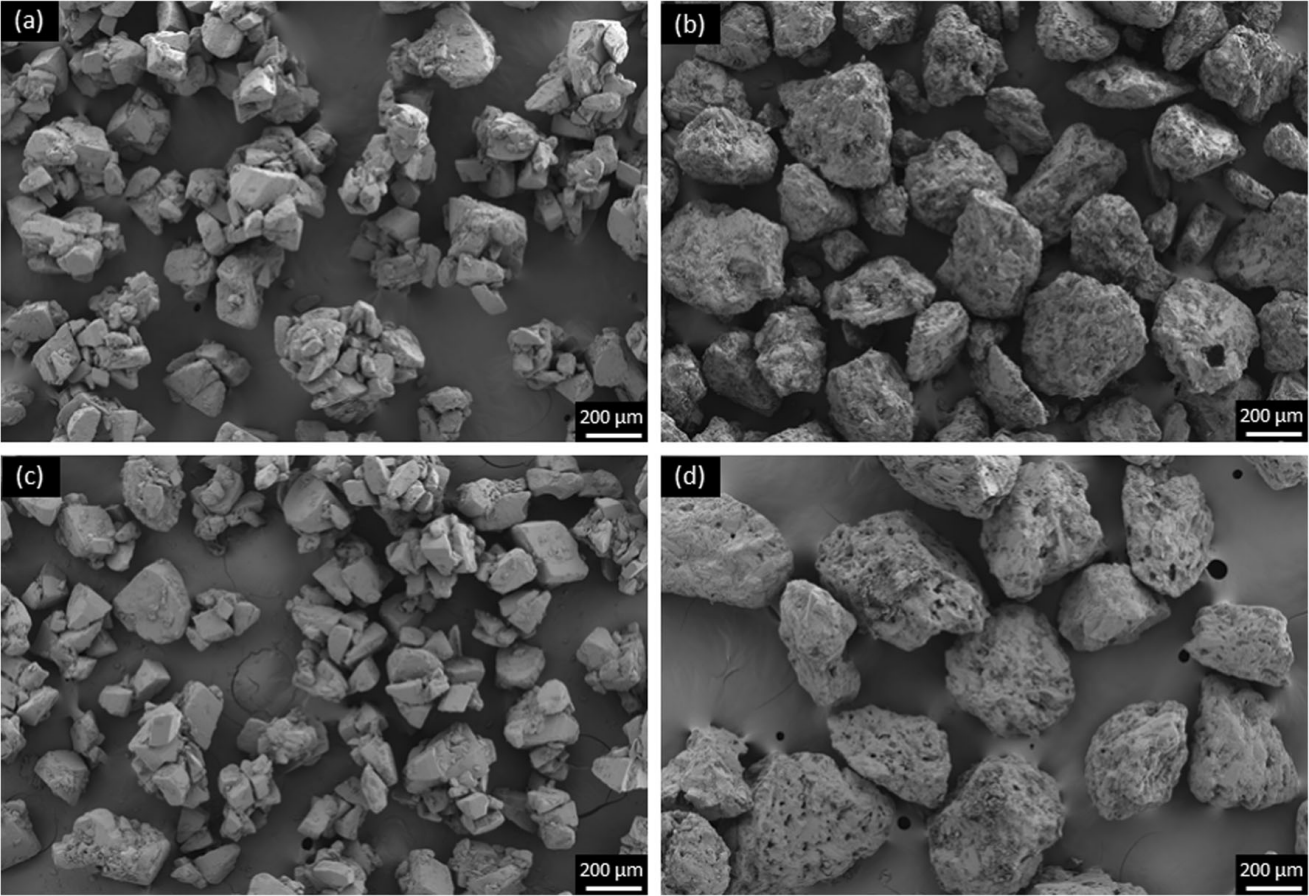
SEM inspection of (**a**) Cap60, (**b**) Pearl300, (**c**) Cap60_WS and (**d**) Pearl300_WS.

Excipient particles with a mean size < 10 μm have the potential to reach the large and small respiratory airways (17) and have shown to be beneficial for the aerosolization performance when present in carrier-based DPI formulations (3, 18). Consequently, it is important to precisely control the excipient particles within this size range (19). As it can be observed in Fig. 3, Pearlitol 300 DC (Pearl300) was composed of large rough particles with a notable quantity of smaller particles filling its crevices and pores. Additionally, laser diffraction (Table I) showed that this D-mannitol grade also presented a preeminent fraction of particles < 10 μm (2.08%). Thus, to engineer particles with a narrower particle size (i.e. by lowering the percentage of fines inherited from primary manufacture) wet-sieving was employed. This allowed to control the amount of fines, by specifically adding a defined mass of them to the coarser excipient particles afterwards. CapsuLac® 60 (Cap60), containing sieved tomahawk α-LH crystal agglomerates (contrary to smoother single tomahawk particles traditionally used as carriers for DPIs) was selected as a control for comparison with D-mannitol (20). In a first step, an anti-solvent screening was carried out (including acetone, isopropanol, n-heptane and decafluoropentane) to identify the most promising solvent with respect to surface smoothing of the carriers (data not shown). Acetone showed to be the most promising solvent and was therefore selected for the present study.

**Table I:** Particle Size Distribution of the Powders (*n* = 3, Mean ± SD)

Sample	Dv_0.1_ (μm)	Dv_0.5_ (μm)	Dv_0.9_ (μm)	SPAN	D_sv_ (μm)	fines < 10 μm (%)
Cap60	112.1 ± 3.8	228.8 ± 11.0	358.2 ± 16.9	1.08 ± 0.01	93.44 ± 5.50	0.47 ± 0.05
Cap60_WS	133.2 ± 1.5	241.9 ± 5.8	428.3 ± 67.9	1.21 ± 0.24	125.68 ± 3.88	0.17 ± 0.01
Pearl300	193.3 ± 6.8	341.5 ± 6.7	514.3 ± 7.7	0.94 ± 0.02	58.07 ± 2.77	2.08 ± 0.10
Pearl300_WS	200.0 ± 0.8	327.9 ± 1.7	462.8 ± 2.8	0.77 ± 0.05	150.12 ± 11.78	0.45 ± 0.01
Pearl300_SC	31.1 ± 1.0	94.1 ± 2.9	208.2 ± 2.9	1.66 ± 0.06	31.99 ± 1.03	1.88 ± 0.04

Prior to wet-sieving, Cap60 did not contain a large fraction of particles < 10 μm, notwithstanding this fraction was lowered from 0.47% to 0.17% (Table I). Wet-sieving of Cap60 also had an impact on the overall PSD of the carrier, in particular the Dv_0.9_. Although, after sieving, a large standard deviation (SD) was found for the Dv_0.9_, a trend indicating an increase of this parameter was still, possible to identify. We hypothesize that this might have been due to the fact that with washing, single particles of Cap60 with a size <100 μm might have been washed away, leaving larger agglomerates of α-LH at the top of the sieve tower. This resulted in a shift of the overall PSD to larger sizes, including the sauter mean diameter (D_sv_). Visual inspection of the carrier via SEM (Fig. 3), before and after washing, did not reveal any notable morphological changes. Wet-sieving of Pearl300 significantly decreased the percentage of fine particles (from 2.08% to 0.45%). The latter, translated into a slight increase of the Dv_0.1_. Although, we fractionated the larger faction of particles of D-mannitol (> 100 μm), there was a notable decrease of the Dv_0.5_ and Dv_0.9_. Due to the fines, which were filling up the pronounced crevices and pores on the surface of Pearl300 particles, being washed away, it can be observed that wet-sieving of D-mannitol resulted in particles with a pumice appearance (Pearl300_WS in Fig. 3). Accordingly, the rough surface of Pearl300 became apparent. Thus, we hypothesize that the pronounced irregularities at the surface of Pearl300_WS led to higher-angle scattering (21), posing an explanation why smaller particle sizes (Dv_0.5_ and Dv_0.9_) were observed after washing. This was also supported by an increase in the surface to volume ratio (D_sv_) (more roughness larger surface).

**Fig. 4:**
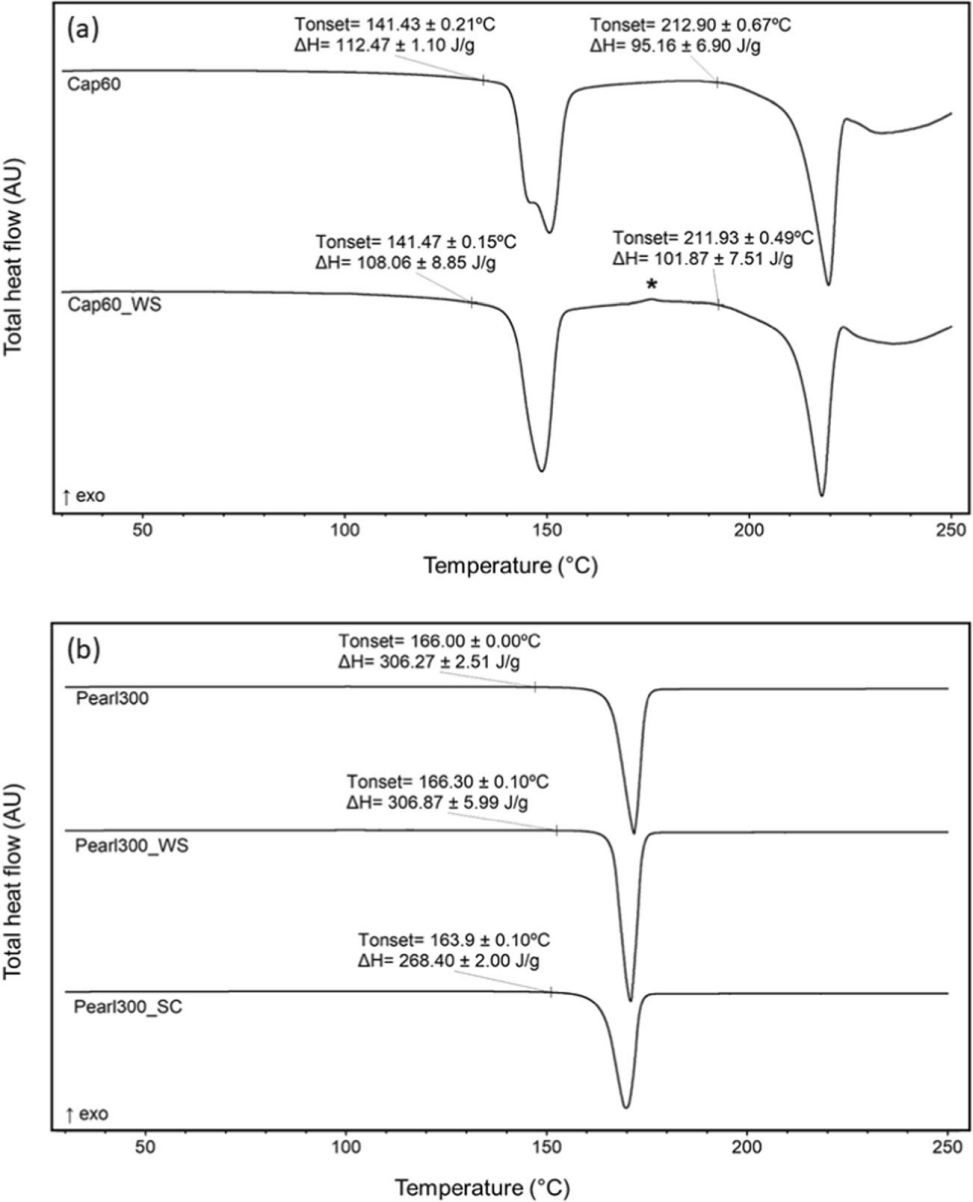
MDSC analysis of the powders (*n* = 3, asterisk indicated exothermic event identified at about 175°C).

**Fig. 5:**
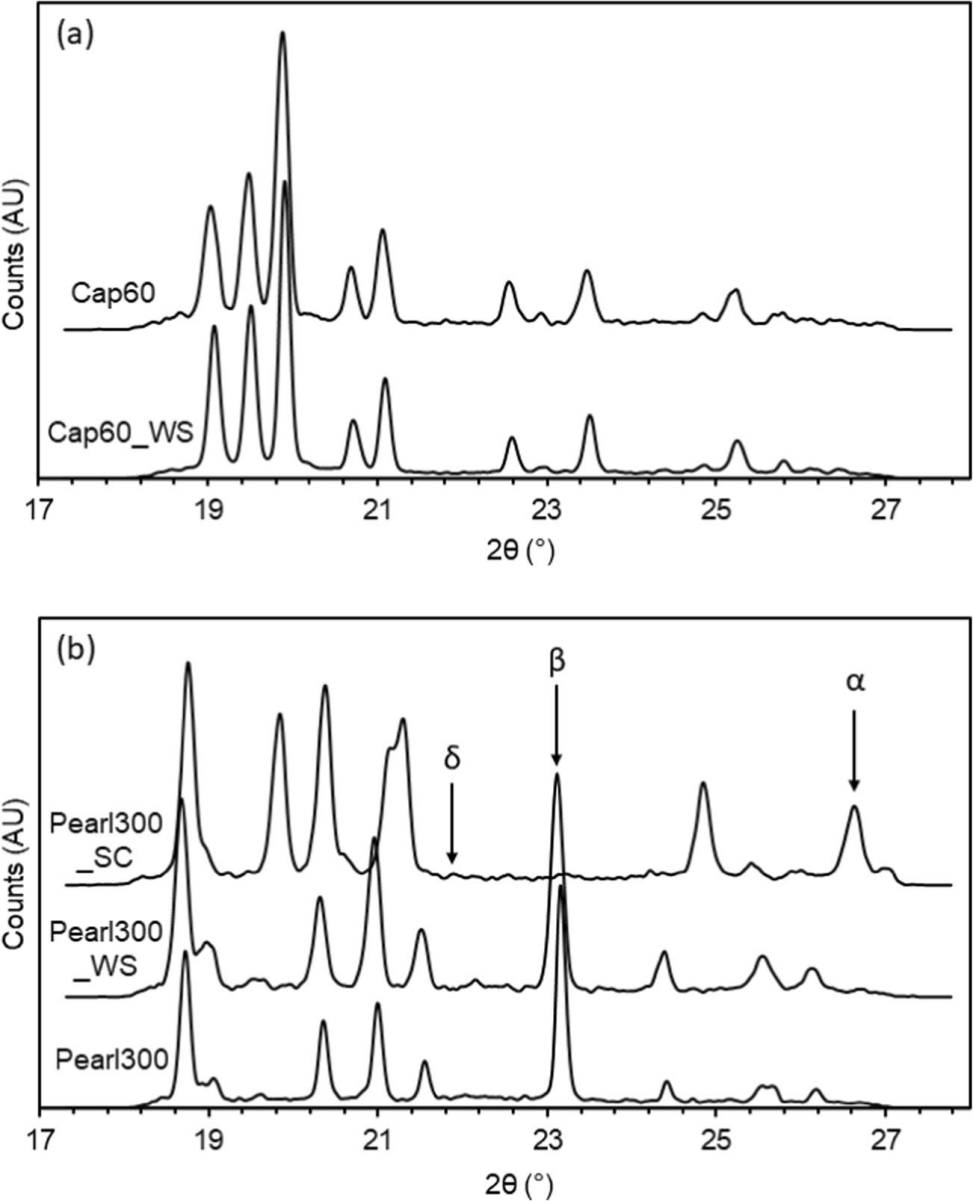
WAXS analysis of the powders (*n* = 3).

Thermal analysis of the samples (Fig. 4) showed that Cap60 had two endothermic peaks; one at ≈ 141°C and another at about 213°C. These peaks were attributed to the dehydration and melting of the α-form, respectively (22). WAXS characterization (Fig. 5) of Cap60 supported the MDSC analysis and confirmed that Cap60 was composed of α-LH, as shown by its characteristic peak patterns at 19.1°, 19.5° and 19.9° 2θ (23). After washing with acetone, the MDSC of Cap60_WS showed narrowing of the dehydration peak and the appearance of an exothermic event at ca. 175°C. This exothermic event was attributed to the unstable form of anhydrous α-lactose (Lα_H_) converting into the anhydrous stable α-form (Lα_s_) at 175°C (24). It is hypothesized that the changes, observed during the calorimetry measurements, were due to acetone impacting the dehydration mechanism of α-LH, leading to the formation of Lα_H_ at higher temperatures (> 130°C) (24, 25). The WAXS patterns of Cap60_WS were consistent with the ones of α-LH, evidencing that at room temperature no polymorphic changes could be detected. MDSC analysis of Pearl300 revealed a single endotherm at 166°C. Due to their very similar melting points the α- and β-forms of D-mannitol are challenging to distinguish when solely using MDSC (26). Thus, by supporting the analysis with WAXS, it was possible to verify the presence of a preeminent peak at 23.1° 2θ, showing that Pearl300 was composed of β-mannitol particles (26). Washing with acetone did not result in any detectable changes of the solid state of Pearl300 (i.e. Pearl300 and Pearl300_WS presented very similar thermograms and WAXS patterns).

Cap60 presented a true density of 1.539 g/cm^3^ (Table II), consistent with known value for α-LH (1.535–1.540 g/cm^3^) (27) and remained unchanged following wet-sieving. Pearl300 and Pearl300_WS showed a true density of about 1.49 g/cm^3^, in line with the known value for the crystals of β-mannitol (26). Regarding the SSA determined by gas absorption (SSA_BET_), Cap60 showed a smaller mean area when compared to Pearl300, i.e., 2798 and 3932 cm^2^/g, respectively (Table II). This, was supported by the observations via SEM (Fig. 3) showing that Cap60 was composed of smooth tomahawk agglomerates and Pearl300 of rough, irregular single particles with deep pores. Wet-sieving of the α-LH carrier (Cap60_WS) resulted in a slight decrease of the SSA (2121 cm^2^/g). Surprisingly, washing with acetone did not result in any change of the SSA of Pearl300. The SSA determined by gas adsorption is related to the nano- and mesoporosities presenting a mean diameter between 1.7 and 300 nm. It is apparent that for the smoother particles of Cap60, the presence of fines with size < 10 μm resulted in an increase of the porosities between 1.7–300 nm. For the very rough particles of Pearl300, the same was not observed. The theoretical specific area (SSA_SV_), was calculated based on the D_sv_, under the assumption that the samples are composed of spherical solid particles, therefore the internal porosities and irregular shapes are not accounted for (15). Thus, the SSA_sv_ values are much lower than the ones obtained by gas absorption. The BR is a parameter often used to evaluate the morphology of carrier particles intended for inhalation (14, 28). It was observed that for Cap60, wet-sieving (Cap60_WS) did not result in any significant changes in the BR (Table II). However, Pearl300_WS showed a BR almost 3 times higher than the one calculated for the raw material. This was in line with SEM observations (Fig. 3), where rougher, pumice single particles of D-mannitol were visualized after wet-sieving.

**Table II:** True Density, Specific Surface Area and Bulk Roughness of the Powders

Sample	ρ (g/cm^3^)^*^	SSA_BET_ (cm^2^/g)^**^	SSA_sv_ (cm^2^/g)†	BR†
Cap60	1.539 ± 0.001	2798.0 ± 38.0	417.1	6.71
Cap60_WS	1.540 ± 0.000	2121.0 ± 36.0	309.9	6.84
Pearl300	1.494 ± 0.000	3932.0 ± 29.0	691.7	5.68
Pearl300_WS	1.489 ± 0.001	3934.0 ± 15.0	268.5	14.65
Pearl300_SC	1.437 ± 0.000	1621.0 ± 51.0	1304.8	1.24

* *n* = 3, mean ± SD, ** mean ± SD of one run

† No standard deviation as parameters were calculate from average values.

For powder handling and formulation, rheological properties like cohesion and flowability obtained from shear cell experiments are important indicators. Comparing these values between the starting materials and the wet-sieved ones it was possible to see that, according to Jenike classification (ffc values between 2 and 4), generally, all powders were cohesive (Table III). Compared to Cap60, Cap60_WS showed slightly lower values of Coh and ffc. For the AIF_eff_ a larger difference was observed and the wet-sieved powder presented a notable smaller value. For Pearl300, the surface treatment notably decreased the Coh, AIF_eff_ and ffc. The AIF supplies information about the frictional forces within the powder (particle-particle) during flow. The friction within the powder is known to be influenced by particle size and shape (29). Considering that wet-sieving did not notably modify the overall shape of the α-LH and D-mannitol particles (Fig. 3), we hypothesize that the differences in the AIF_eff_ were mostly driven by washing out the particles <100 μm. Hence, we purpose that the fractionation of the powders led to the reduction of particle-particle contacts, decreasing friction within the powder (30). As a result of reduction in powder friction, slightly lower cohesion and ffc values were found indicating a potential improvement in flowability. For Pearl300, wet-sieving led to more notable changes in the particle size, and consequently a larger difference in powder flow values.

**Table III:** Cohesion, Angle of Internal Friction and Flowability Factor of the Powders (*n* = 3, mean ± SD)

Sample	Coh (kPa)	AIF_eff_ (°)	ffc
Cap60	1.96 ± 0.22	27.13 ± 2.30	2.57 ± 0.26
Cap60_WS	1.37 ± 0.38	22.07 ± 1.56	3.53 ± 0.68
Pearl300	2.29 ± 0.78	35.07 ± 2.19	2.21 ± 0.52
Pearl300_WS	1.16 ± 0.10	22.07 ± 1.56	4.07 ± 0.19
Pearl300_SC	0.27 ± 0.06	23.50 ± 0.28	15.87 ± 4.03

### Particle Engineering by Spray-Congealing

Since α-LH degrades upon melting (31), it was only possible to engineer Pearl300 through spray-congealing. During processing no evidence of any yellowish sample, characteristic of the degradation of sugars due to oxidation, was observed (32), thus it was inferred that D-mannitol was stable enough to be processed via spray-congealing.

**Fig. 6:**
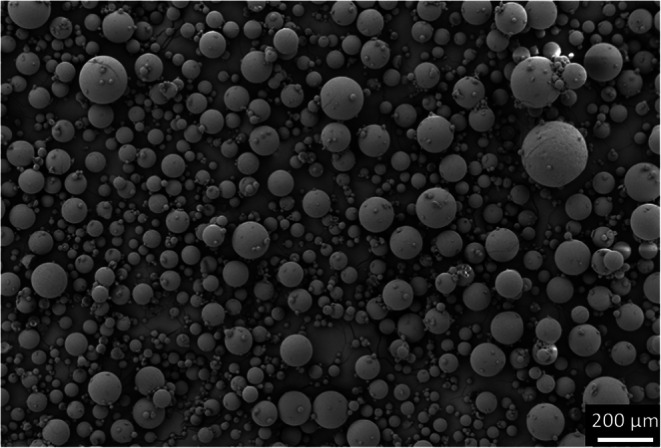
SEM inspection of Pearl300_SC.

SEM images in Fig. 6 show that spray-congealing resulted in spherical particles. The spray-congealed particles were also notably smaller than their raw and wet-sieved counter-parts, presenting lower Dv_0.1_, Dv_0.5_ and Dv_0.9_. Additionally, they also presented a larger SPAN when compared to the other D-mannitol samples. Indeed, in Fig. 6, it is possible to observe the presence of single spherical particles of diverse sizes. During spray-congealing, by the use of a twin-fluid nozzle, the viscous liquid (melt) was injected into a high velocity gas leading to the formation of droplets (33). Thus, when compared to other melt solidification techniques, i.e., prilling, where large narrowly monodispersed particles are obtained due to the use of nozzles that rely on hydrodynamic instabilities (34), twin-fluid atomization led to the formation of smaller particles. Additionally, it is speculated that because jet-breakage is more uncontrollable, droplets of various sizes were formed, leading to solid particles in a broader size span. Likewise, the Pearl300_SC sample also showed a considerable amount of fine particles below 10 μm (1.88%).

MDSC analysis (Fig. 4) of Pearl300_SC showed an endothermic event with a lower onset temperature (164°C) and enthalpy (288.40 J/g), when compared to the raw material. As mentioned before, from MDSC it is hard to distinguish between D-mannitol polymorphs; however, X-ray patterns of the α, β and δ forms of D-mannitol present very distinct Bragg peaks that allow their distinction. The α, β and δ forms are characterized by the peaks at 26.6°, 23.1° and 21.9°2θ, respectively (26). From WAXS patterns in Fig. 5, it is possible to distinguish a clear peak at 26.66° 2θ and a very small one at 21.9° 2θ. No peak could be found at 23.1° 2θ. Thus, it was concluded that Pearl300_SC was predominately composed of the α-form of D-mannitol with trace amounts of the δ-form. Although, Pearl300_SC presented a homogenous and smooth surface topography typical of non-crystalline surfaces, MDSC and WAXS of the same sample clearly indicate that the material was predominantly crystalline.

Spray-congealing slightly decreased the true density compared to the starting and wet-sieved materials (Table II), potentially, due to the pure α-form presenting a lower true density (1.468 g/cm^3^) (26). Further, given that after melting the α particles crystallized with the δ polymorph as an impurity, it is possible that the δ-mannitol acted as a crystal habit modifier affecting the true density of the compound (35). However, more investigations would be needed to confirm this. Compared to Pearl300 and Pearl300_WS, the spray-congealed samples show a significantly reduced surface area (SSA_BET_) and roughness (BR). In contrast to all other non-spherical carriers, the SSA_sv_ for Pearl300_SC is comparable to the SSA_BET_. Besides the spherical shape in line with the assumptions made for the SSA_sv_ calculation, this also indicated that Pearl300_SC does not present notable morphological irregularities or/and porosities.

Pearl300_SC showed notably smaller values of cohesion and a larger ffc (Table III), when compared to the raw and wet-sieved materials. Engineering by spray-congealing changed the D-mannitol powder from cohesive to free flowing. Interestingly, the AIF_eff_ value of the Pearl300_SC and Pearl300_WS were very similar, thus it is more likely that the improvement in flowability is due to the packing state of the powder bed. It is assumed that due to the spherical and smooth nature of Pearl300_SC particles, the powder will present similar density in its packed and dilated conditions (Hausner ratio), facilitating the powder flow (36).

The presence of trace amounts of δ-mannitol in Pearl300_SC raised concerns about the stability of the particles hence, the sample was conditioned for 24 h at 93% RH (set with saturated salt solution of potassium nitrate in a sealed chamber). Following the latter, it was determined that the δ-form was successfully converted into the more stable β-mannitol (Supplementary Material Fig. S1), and mixing and aerosolization experiments were carried out with this sample.

### Impact of Particle Engineering on DPI Performance

**Fig. 7.**
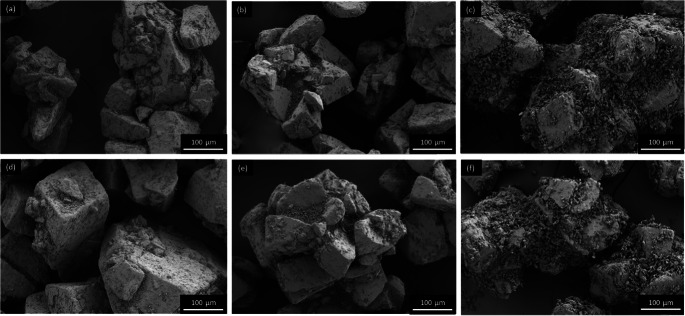
SEM images of adhesive mixtures with Cap60: (**a**) + BDP (**b**) + MgSt + BDP (**c**) + Preblend + BDP and Cap60_WS (**d**) + BDP (**e**) + MgSt + BDP (**f**) + Preblend + BDP.

**Fig. 8:**
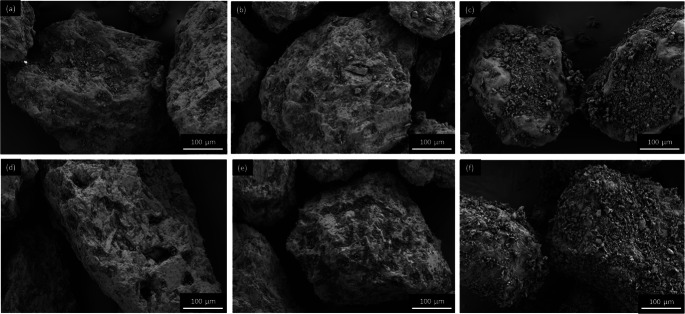
SEM images of adhesive mixtures with Pearl300: (**a**) + BDP (**b**) + MgSt + BDP (**c**) + Preblend + BDP and Pearl300_WS (d) + BDP (**e**) + MgSt + BDP (**f**) + Preblend + BDP.

**Fig. 9:**
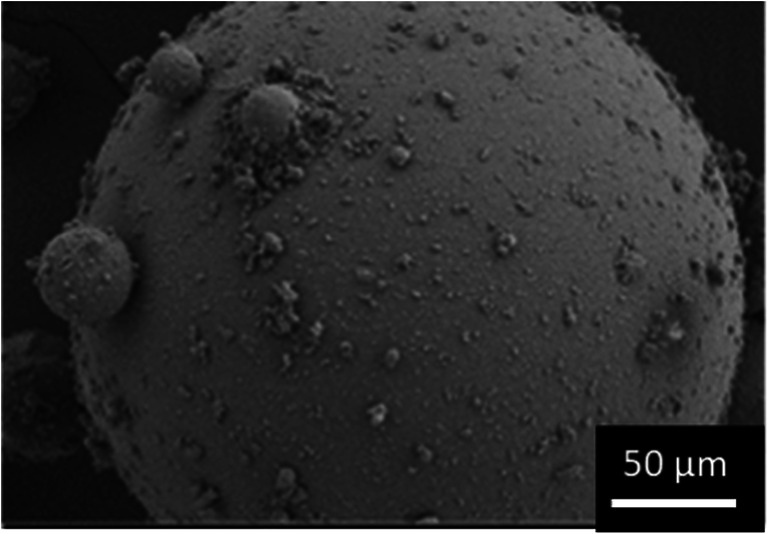
SEM image of Pearl300_SC + BDP.

All carriers (starting material, wet-sieved and spray-congealed ones) were blended with 1 wt% BDP (binary mixtures) and additionally, with a controlled amount of fines, namely 0.2 wt% MgSt and 10 wt% of ‘Preblend’ (fine α-LH + MgSt) (ternary mixtures). It is, generally, reported that rough and irregular carrier particles have a higher loading capacity compared to smooth ones (37). In more rough/irregular particles, the higher number of indentations and cavities present, allow a greater number of fine particles to fill and/or coat the surface of the carrier. Likewise, with the very smooth uniform Pearl300_SC no ternary blends were prepared as the surface morphology (smooth and uniform, without any cavities and a low surface area) was not expected to have a high loading capacity (38). After mixing all the blends presented an RSD < 7% (Table IV). According to Hassan *et al*., blends with an RSD below 10% are considered homogenous (39), thus we found the mixtures appropriate to be further tested concerning their *in vitro* aerodynamic performance. Representative SEM images of the blends are presented in Figs. 7, 8 and 9 (and Supplementary Material Fig. S2–4).

**Table IV:** Mixing Homogeneity of the Binary and Ternary Blends (*n* = 10)

Raw materials	BDP RSD (%)	Engineered materials	BDP RSD (%)
Cap60	3.4	Cap60_WS	6.9
Cap60 + MgSt	6.5	Cap60_WS + MgSt	6.4
Cap60 + MgSt + Preblend	1.9	Cap60_WS + MgSt + Preblend	6.3
Pearl300	3.0	Pearl300_WS	3.0
		Pearl300_SC	1.1
Pearl300 + MgSt	6.2	Pearl300_WS + MgSt	4.9
Pearl300 + MgSt + Preblend	6.1	Pearl300_WS + MgSt + Preblend	2.4

#### Impact of Particle Engineering on Shot Weight Consistency

The shot weight consistency is a crucial factor to ensure that the release of every dose is the same and therapeutic outcomes are met (40). Therefore, two different inhalers of each blend were tested concerning the consistency of 10 consecutive shots and the inter- and intra-device variability were evaluated.

The NEXThaler® is a breath-actuated reservoir device in which a powder is first precisely dosed by gravity into a recess and then de-agglomerated in a vortex chamber. To evaluate the dose-mass consistency, it is important to understand how the particle properties of different carriers might have affected these two steps.

**Fig. 10:**
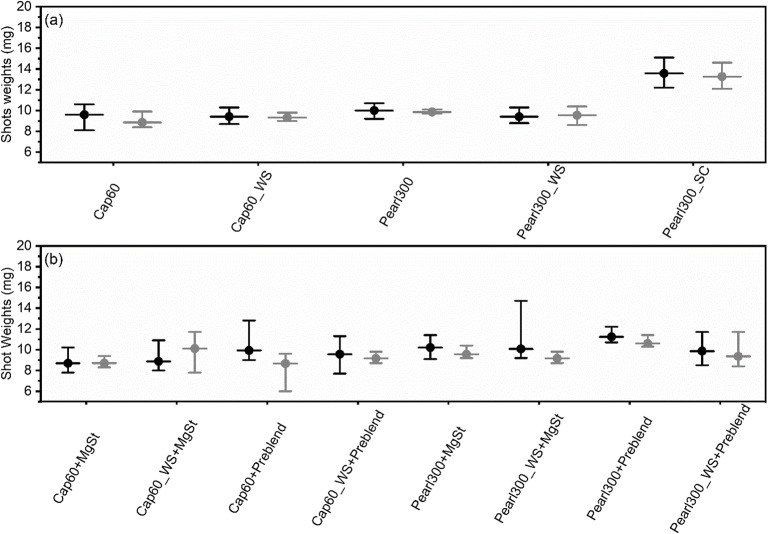
Shot weight consistency for: (**a**) binary blends with BDP (**b**) ternary blends with BDP, MgSt and ‘Preblend’. Black and grey dots represent one inhaler each (*n* = 10, mean, maximum and minimum value of the shot weights are represented).

In Fig. 10(a), it can be observed that for the binary blends, engineering of Cap60 and Pearl300 by wet-sieving led to lower inter-device variability. We propose, this was a consequence of the more uniform sized powders (lower SPAN) produced via wet-sieving. Due to the disparity between device performances containing Pearl300 as a carrier, no clear inference concerning the effect of wet-sieving on intra-device variability could be made. For Cap60, the narrower PSD of the wet-sieved material also seemed to beneficially affect intra-device variability. A crucial observation was that spray-congealing of Pearl300 led to a higher mean mass of powder being dosed per shot (increase from about 9.9 to 13.4 mg). We hypothesize, that this was mainly related to the spherical shape of the spray-congealed material. Spherical shaped materials are known to pack more uniformly compared to irregular ones, so for the same recess volume a higher powder mass could be dispensed (41, 42). Additionally, it was observed that for Pearl300_SC the two devices tested showed extremely identical performances. Here, this cannot be attributed to the SPAN of the material as Pearl300_SC actually, showed a higher value compared to Pearl300. However, Pearl300_SC demonstrated better flow when compared to the raw and wet-sieved materials. Improvement in flow has been associated with more consistency during powder filling (42) and could potentially explain the observed results. Still, Pearl300_SC showed a larger intra-device variability (RSD ~ 6%). During vortex fluidization, particles of distinct sizes accelerate at different speeds, possibly leading to segregation; i.e., smaller lighter particles leave the device more readily and larger and heavier ones will have more tendency to move inwards (43). Hence, the greater intra-device variability could be associated to the larger differences in PSD of Pearl300_SC.

The addition of MgSt (Fig. 10(b)) to the blends of the raw materials and BDP (Cap60 + MgSt and Pearl300 + MgSt) did not have any notable impact on the inter- and intra-device variability (Fig. 10(b)). However, for the wet-sieved materials the use of the salt led to a pronounced deterioration of inter- and intra-device variability (Cap60_WS + MgSt and Pearl300_WS + MgSt). The reason underlying this behavior needs to be clarified; however, it is known that MgSt preferentially fills large cavities of coarser particles, and its optimal effect on flow follows a non-linear correlation with the radius of the particles to be lubricated (44, 45). Considering the differences induced by wet-sieving in the surface topography and micromeritic properties (i.e., BET, PSD, roughness) of α-LH and D-mannitol, it is not entirely surprising, that distinct impacts on powder flow were observed when using the same concentration of MgSt on the raw and wet-sieved materials. In future work, the effective concentration of MgSt to improve the performance of carrier particles with distinct characteristics will have to be investigated.

The addition of ‘Preblend’ to Cap60 and Cap60_WS with BDP led to higher differences in intra- and inter-device variability (Fig. 10(b)). For Pearl300, the addition of ‘Preblend’ increased the mass of the emitted dose (from about 9.5 to 10.9 mg) and improved inter-device variability. Whereas, for Pearl300_WS, the addition of ‘Preblend’ did not have any notable impact on inter-device variability, but increased the intra-device RSD from ~4.5% to 9.2%. We hypothesize, that the different impact of ‘Preblend’ on α-LH and D-mannitol carriers was related to the very distinct surface topography of the two carriers. D-mannitol carriers were considerably more corrugated than the α-LH ones and, thus expected to be able to host more fine excipient particles onto/into their surface (46). On the contrary, the less corrugated two α-LH materials (Cap60 and Cap60_WS) could not accommodate the fines as efficiently, leading to segregation (between coarse carrier particles hosting fines and particulate agglomerates of fines, see supplementary material Fig. S2 (c) and (f)) (37). Thus, powder flow was deteriorated and an increase in inter- and intra-device variability was seen. For Pearl300, it is speculated that the concentration of ‘Preblend’ plus the initial fines content of the carrier were ideal to adequately coat its rough surface, originating a denser packing, that produced an increase of the emitted mass per shot. In contrast, for the rougher surface of Pearl300_WS with reduced initial amount of fines, the concentration of ‘Preblend’ was not enough to cause potential changes in the powder bed packing; thus, no differences in the emitted mass per shot were observed. With respect to intra-device variability, it is suggested that the more efficient detachment of fine particles from Pearl300_WS than from Pearl300 (shown by its higher FPF in Fig. 13) also resulted in a more efficient de-agglomeration; this, in turn leading to more particles of different sizes within the vortexes and higher dose variability (as already explained for the Pearl300_SC blends).

### Impact of Particle Engineering on *In Vitro* Inhalation Performance

The ED, FPM, and FPF were used to evaluate the performance of engineered D-mannitol particles in comparison to the D-mannitol reference material as well as α-LH engineered and starting material in binary and ternary blends. No pronounced differences could be detected in the MMAD among the different formulations. All the blends presented an MMAD of 1.8 ± 0.5 μm.

#### Impact of Particle Engineering and Addition of Ternary Agents on the Drug Dose

**Fig. 11:**
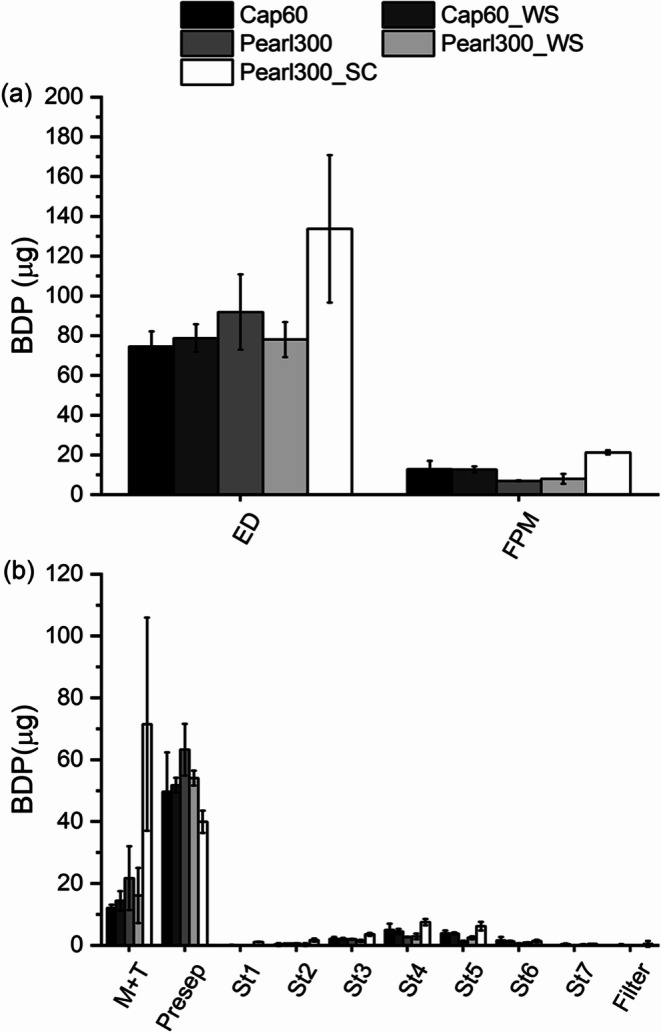
(**a**) Emitted dose (ED) and fine particle dose (FPD) as well as (**b**) stage-wise deposition for the binary blends of starting, wet-sieved and spray-congealed materials (*n* = 2, mean ± range).

Looking first at the binary blends, the ED values were comparable between starting and wet-sieved materials (Fig. 11(a)) and no notable differences were found between D-mannitol and α-LH particles. Further, this was in line with the shot weight results where no difference in emitted mass was observed. The FPM was slightly lower for both, wet-sieved D-mannitol and α-LH. By contrast, Pearl300_SC showed 75% higher ED and 168% higher FPM related to Pearl300. Concerning the FPF of the various D-mannitol carriers (Fig. 13), a slight increase in the FPF was observed when using Pear300_SC, indicating better API de-attachment. Hence, it can be inferred that the improvement in ED and FPM was mainly due to two factors; the better API de-attachment and the increase in the mass delivered per shot (about 40% more), as explained in the previous section.

Comparing the stage-wise deposition of the binary blends (Fig. 11(b)), it can bee seen that the wet-sieved and starting materials showed comparable deposition. Only the use of Pearl300_WS slightly reduced the amount of drug deposited in the mouth and throat (M + T) and pre-separator (Presep) and increased the amount of drug on stage 5. The deposition pattern of Pearl300_SC was different from the other binary blends and a very high amount of API was found in the M + T. This led to the assumption that drug detachment is quite efficient, even taking place too early and allowing for loose API to be deposited already in the mouth and throat (not being transported to the deep lung). Further, higher amounts of API were deposited on stage 3, 4 and 5. Again, the higher shot weight and resulting larger amount of BDP per NGI experiment could have, in part, contributed to this.

**Fig. 12:**
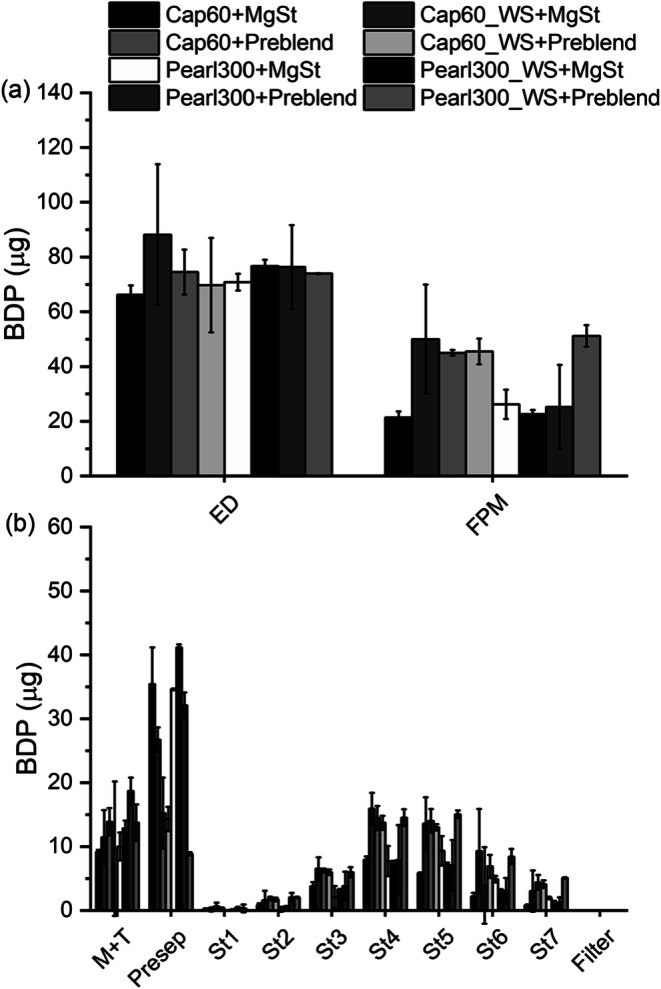
(**a**) Emitted dose (ED) and fine particle dose (FPD) as well as (**b**) stage-wise deposition for the ternary blends of starting and wet-sieved materials (*n* = 2, mean ± range).

**Fig. 13:**
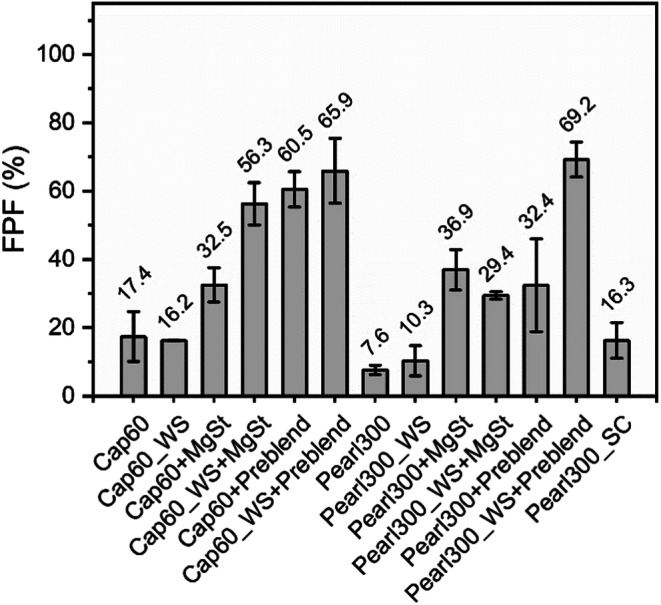
Fine particle fraction (FPF) of the binary and ternary blends of starting and engineered α-LH and D-mannitol carrier materials (*n* = 2, mean ± range).

The addition of ternary agents is reported to increase the deposition of the API at the lower stages of the NGI by varying mechanisms (19, 47). Likewise, it was not surprising that in the present study, the addition of MgSt and ‘Preblend’, overall, increased the FPM for D-mannitol and α-LH carriers (binary vs ternary blends, Fig. 11(a) and 12 (a), respectively); while the ED remained comparable. Looking at the stage-wise deposition in Fig. 12(b) and comparing it with the binary blends in Fig. 11(b), the addition of MgSt increased the amount of BDP on stage 4 and 5 of the impactor and limited the amount in the Presep (this was true for α-LH and D-mannitol, wet-sieved and starting material). MgSt is used as a force control agent in DPI formulations in order to reduce the adhesive interactions of the API with the carrier surface, improving de-attachment (48). Likewise, in the present study the use of the salt, led to more API being found in the lower stages of the impactor and higher FPFs (Fig. 13). The improvement in FPM was even more pronounced when ‘Preblend’ was added to Cap60 and Pearl300_WS. Interestingly, Cap60_WS was superior compared to Cap60 in combination with MgSt (lower deposition in Presep and higher deposition in stages 3 to 7) and Pearl300_WS was superior over Pearl300 in combination with ‘Preblend’ (lower deposition in Presep and higher deposition in stages 4 to 7).

Given that, excluding Pearl300_SC, all the blends presented very similar EDs, the differences in FPM can, generally, be attributed to how drug de-attachment was affected by engineering and/or the addition of ternary agents.

#### Impact of Particle Engineering and Addition of Ternary Agents on Drug de-Attachment

Figure 13 compares the FPF of all binary and ternary blends. Here, one can clearly see how the different carriers and ternary agents affected drug de-attachment. The highest FPF of all the tested formulations was achieved using Pearl300_WS in combination with ‘Preblend’. For this system, the wet-sieved carrier showed a notably better performance compared to the starting material. This can be explained by the deep voids and clefts on the surface of Pearl300 revealed during the wet-sieving process. It is proposed that the latter were filled up with ‘Preblend’ during the blending process and allowed BDP to be more efficiently detached from the wet-sieved D-mannitol carrier surface (49). In contrast, the comparably smaller amount of MgSt was not enough to cause this effect. For Cap60, the addition of ‘Preblend’ resulted in the same FPFs for starting and wet-sieved materials. Here, compared to the starting material, the wet-sieved sample only showed an increase in FPF when MgSt was added. This can be explained by the morphological difference of the starting materials (α-LH vs D-mannitol). Cap60 showed no voids and clefts, where the API could be sheltered from aerosolization forces. Moreover, it is hypothesized that, here, the reduction of initial fines (0.49%) to 0.17% by wet-sieving also played a crucial role when a small amount of ternary agent (0.2% of MgSt) was added. MgSt has a high affinity to bind to lactose (no electrostatic repulsion) (50). Thus, it is postulated that the addition of MgSt to Cap60_WS with lower intrinsic α-LH fines, successfully covered the ‘active sites’ (high energy binding sites on the carrier surface) on the carrier surface and forced API particles to interact with passive or low energy sites, promoting its detachment (51). By contrast, when ‘Preblend’ was added, the difference in initial fines content (Cap60 vs Caps60_WS) becomes insignificant and the behavior of Cap60 and Cap60_WS blends is dominated by the high amount of α-LH fines (10%) compared to intrinsic α-LH fines (0.4%7 vs 0.17%). Pearl300_WS, the powder with the highest bulk roughness, showed the best performance when in combination with 10% ‘Preblend’. In turn, Pearl300, was the only carrier where the addition of ‘Preblend’ did not further increase the FPF compared to the ternary blends with MgSt. For Pearl300, the total amount of intrinsic fines plus API seemed already too high, for the addition of ‘Preblend’ to cause a positive effect (13.08% compared to 11.45% for Pearl300_WS, 11.47% for Caps60 and 11.17% for Cap60_WS formulated with ‘Preblend’). A threshold where a certain number of fines, does not further improve or even reduce the aerosolization performance has been reported before (1, 19). Results vary depending on the type of device and formulation used. The difference in type and amount of fines that show a superior effect on the aerosolization performance of α-LH and/or D-mannitol is most likely connected to the effect that different mechanisms (active site, buffer, agglomeration and fluidization theories) have on API detachment. These effects might be different for the two materials and dependent on the surface topography and structure of the carrier.

The FPF delivered from the spray-congealed carrier was 16 ± 2% (Fig. 12) and notably higher when compared to the starting material of D-mannitol (FPF = 8%). Comparison of the FPF of Pearl300_SC to the one from the wet-sieved material, shows that the use of Pearl300_WS without ternary agents did not notably impact the FPF of D-mannitol. Likewise, without the addition of ternary agents, spray-congealing of D-mannitol has shown to be promising in terms of the relative increase of the FPF, when in relation to the starting material (around 100%).

### Applicability of the D-Mannitol Particles Engineered by Wet-Sieving and Spray-Congealing

In order to get an idea of the performance of our novel engineered carriers (Pearl300_SC and Pearl300_WS in combination with ‘Preblend’) related to other engineered D-mannitol carriers, the FPF and relative increase in FPF (RI-FPF) for a few selected systems tested with the Novolizer® was compared. The Novolizer® also uses vortex-based fluidization and is considered most similar to the NEXThaler® used in the present study. In our study, the FPF for wet-sieved mannitol with ‘Preblend’ was 69.2%, and the FPF of Pearl300 with ‘Preblend’ 32.4% resulting in a RI-FPF of 114%. Scherließ *et al*. reported for Parteck® M DPI (Merck, Germany), a commercial mannitol product for DPIs, FPF values of around 40% (9, 10). Compared to that the RI-FPF for Pearl300_WS + ‘Preblend’ would be 75%. In the aforementioned study, Parteck® M DPI was tested in blends with ternary agents (MgSt and mannitol fines) and the RI-FPF was 55% (for budesonide) and 65% (for salbutamol sulphate), depending on the API that was used (10). Littringer at al. produced spray-dried mannitol particles and the FPF of salbutamol sulphate varied from 11% to 29% dependent on the surface roughness and topography (indentations) of the carrier material. However, no comparison with the starting material was done (52). Our Pearl300_SC, spherical particles with a smooth surface and slightly larger size showed a FPF of 16% and RI-FPF of 100%. Overall, the FPF or relative improvement of the *in-vitro* aerosolization performance (RI-FPF) of Pearl300_WS + ‘Preblend’ and Pearl300_SC is superior or equal to other reported mannitol carriers. However, we note that direct comparison is not straight forward, as besides the inhalation device, performance is further affected by the carrier size, the API used, formulation and blending procedure and a combination of thereof. Therefore, we caution here that this comparison is just to showcase the relative extent of FPF gained (or not) by a certain route of carrier particle engineering and/or ternary agent addition.

Finally, in the competitive market of inhalable medications, especially for patients with asthma and chronic obstructive pulmonary disease (COPD), there are a multitude of available devices each with different operating principles. Reservoir multi-dose DPI devices such as NEXThaler® and Novolizer® represent the preferred solution in terms of easiness of administration and portability for the chronic therapies, increasing adherence and minimizing errors in device handling. On the other hand, developing a DPI formulation for this kind of devices is, generally, a difficult task. Indeed, the DPI powder must behave with a high level of reproducibility during the whole life of the device. In particular, the challenge is to have good powder flow behavior to ensure the adequate and constant metering of the doses over the period of a device use. This aspect is less challenging in a unit-dose or multi-unit dose device, where a filling machine can be customized to properly meter the single dose with a larger range of viable options. Furthermore, also the de-agglomeration and aerosolization of the metered dose must be adequate in terms of performance and reproducibility, and constant through the whole life of the multi-dose device. For this reason, usually a carrier-based formulation is needed to be combined with a multi-dose reservoir device and very often the formulation platform is co-developed with the device to give a reciprocal adaptation. For this reason, the carrier-based DPI formulation described in this paper, showed noteworthy results. Indeed, these formulations were not specifically co-developed with a reservoir multi-dose device, but only adapted on an existing one, designed for other DPI carrier-based platforms. In particular, as a DPI carrier, the spray-congealed D-mannitol, Pearl300_SC (53), showed to be able to deliver drugs with a good reproducibility of shot weight and without the use of ternary agents such as MgSt. An optimized carrier with similar characteristics could be beneficial to reduce the number of mixing process steps and avoiding the inhalation of excess excipient fine materials. Subsequently, scale-up trials were done in order to evaluate scalability of the process and two 500 g batches were successfully produced with comparable particle characteristics and inhalation performance (53). By optimizing the blending protocol for spray-congealed D-mannitol and, reducing drug deposition in mouth and throat, this engineered material is expected to be a promising candidate as a novel inhalation carrier.

## Conclusion

In our study, we showed the results of spray-congealing and wet-sieving of D-mannitol as innovative particle engineering processes to develop superior inhalation carrier particles. Wet-sieving, being an appropriate technique to generate smooth carriers with a narrow particle size distribution enabled better control of the number of fines needed in the formulation. Results have shown that the wet-sieving process and the related aerosolization performance are strongly dependent on the topography and structure of the starting material. Further, this study, again showed the interdependency of carrier particle surface and formulation parameters and their effect on inhalation performance; α-LH and D-mannitol do not show identical performance when engineered via wet-sieving and the same ternary agents are added. The more uniform pumice particles with deep voids and clefts of Pearl300_WS led to a beneficial effect in combination with 10% ‘Preblend’. By contrast, for the agglomerated Cap60_WS with smooth surfaces and pronounced edges, the addition of 0.2% MgSt was advantageous for the inhalation performance. Spray-congealing, a single step process, has shown its potential in generating smooth spherical particles of δ-D-mannitol that easily (conditioning overnight) can be converted into the stable β-form. The initial benefit of using spray-congealed D-mannitol to enhance the *in vitro* aerosolization performance of binary blends of the carrier with a low dose of BDP was shown (RI-FPF of 100%).

### ACKNOWLEDGMENTS AND DISCLOSURES

This work was funded through the Austrian COMET Program by the Austrian Federal Ministry of Transport, Innovation and Technology (BMVIT), the Austrian Federal Ministry of Economy, Family and Youth (BMWFJ) and by the State of Styria (Styrian Funding Agency SFG). The authors would also like to thank Sabrina Mertschnigg at FELMI-ZFE − Austrian Centre for Electron Microscopy for her assistance in the scanning electron microscopy measurements, Meggle for kindly providing lactose samples, Roquette for providing D-mannitol samples and Bruker AXS GmbH (Germany) for allowing the use of their equipment. TG and FS are employees of Chiesi Farmaceutici; HS is an employee of Austrian Centre for Electron Microscopy and Nanoanalysis; JTP, SZ and AP are employees of the Research Center Pharmaceutical Engineering GmbH, which received funding for their work from Chiesi Farmaceutici.

## Supplementary Information

ESM 1 (DOCX 2753 kb)
