# Supplementary material for: Spray-Congealing and Wet-Sieving as Alternative Processes for Engineering of Inhalation Carrier Particles: Comparison of Surface Properties, Blending and In Vitro Performance
Source: Pharm Res. 2021 Jun 10;38(6):1107–23. doi: 10.1007/s11095-021-03061-5 (PMC8217042; doi:10.1007/s11095-021-03061-5)
Supplement: Supplementary file 1 — (DOCX 2753 kb) [file 11095_2021_3061_MOESM1_ESM.docx]

# Supplementary Material


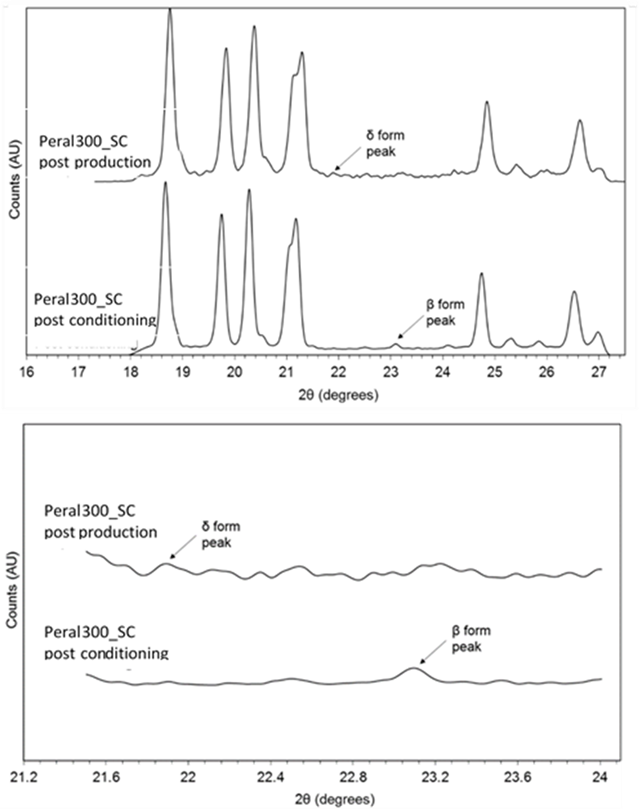


**Figure S1: WAXS analysis of the Pearl300_SC post production and post conditioning**


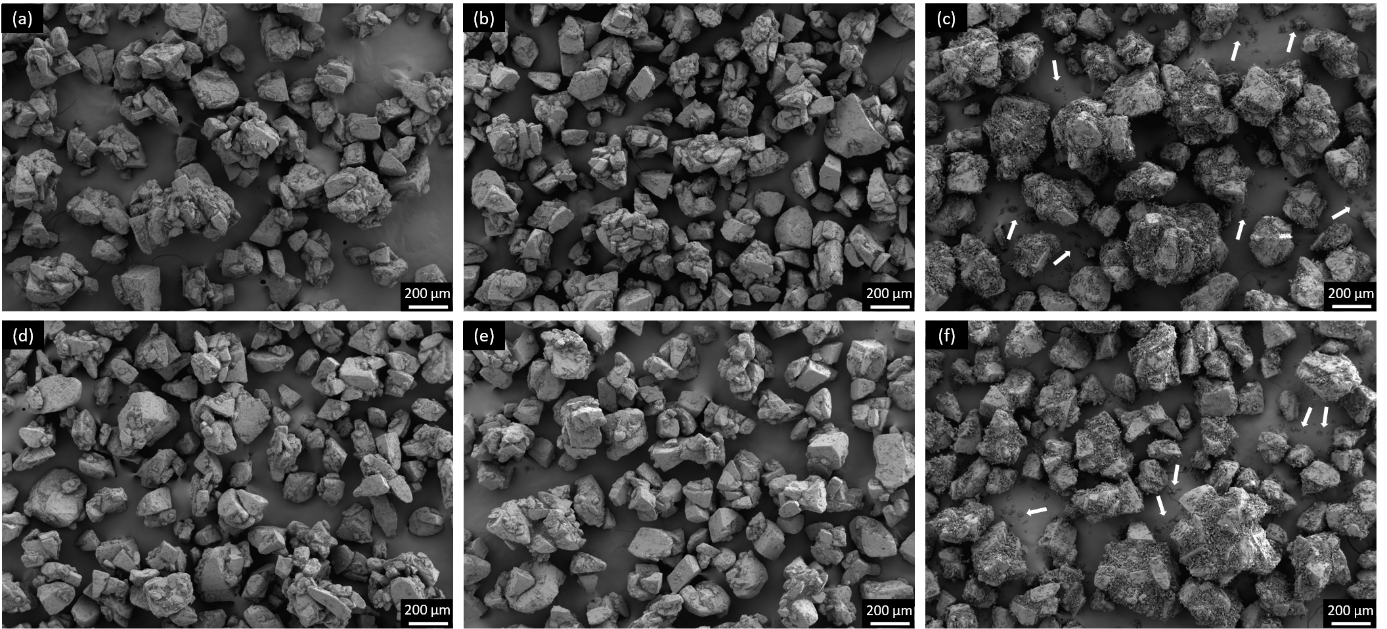


**Figure S2: SEM images of the adhesive mixtures with Cap60: (a) + BPD, (b) + BDP + MgSt, (c) + BDP + MgSt + Preblend and Cap60_WS: a) + BPD, (b) + BDP + MgSt, (c) + BDP + MgSt + Preblend (arrows indicate particulate aggregates detached from the coarse carrier)**


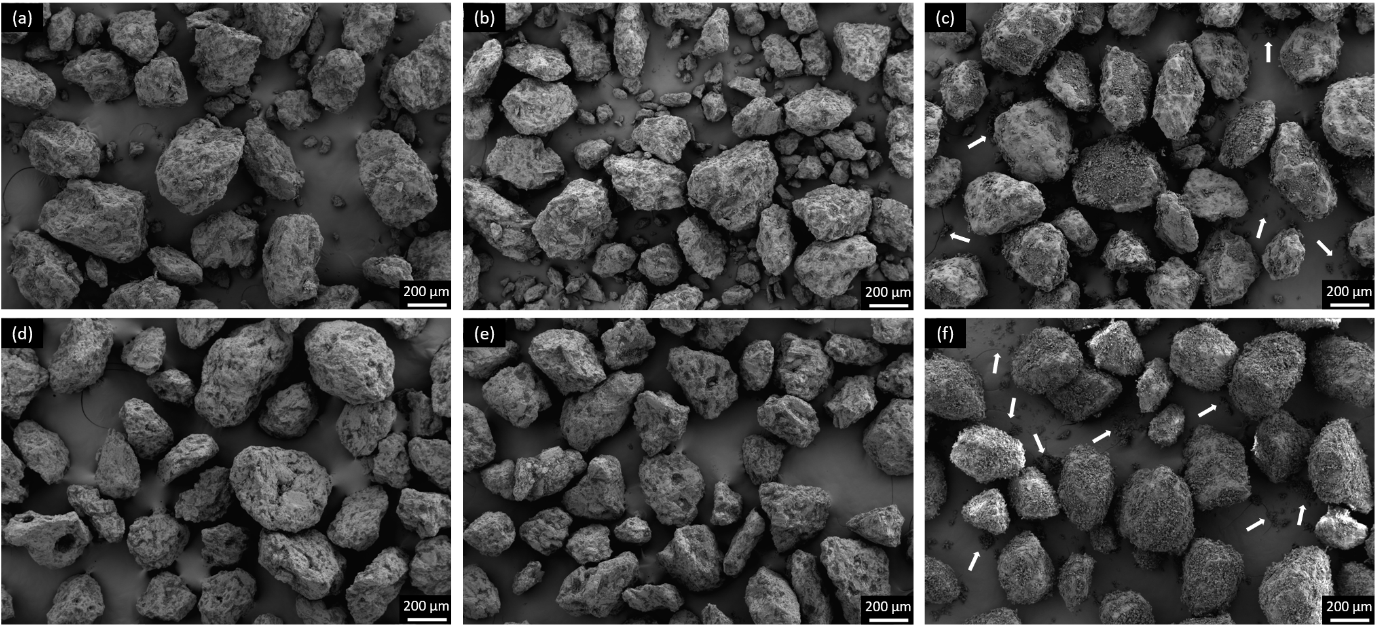


**Figure S3: SEM images of the adhesive mixtures with Pearl300: (a) + BPD, (b) + BDP + MgSt, (c) + BDP + MgSt + Preblend and Pearl300_WS: a) + BPD, (b) + BDP + MgSt, (c) + BDP + MgSt + Preblend (arrows indicate particulate aggregates detached from the coarse carrier)**

**
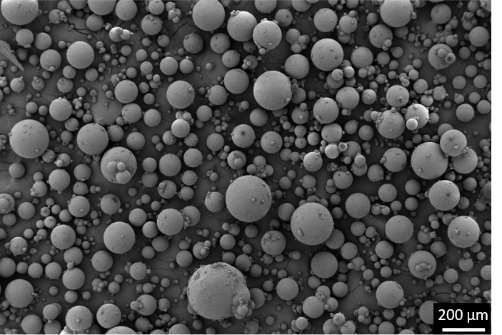
**

**Figure S4: SEM images of the adhesive mixtures with Pearl300_SC + BPD**
